# Physicochemical and Thermal Properties of Aluminosilicate Gels Based on Metakaolin Doped with Different Amounts of Samarium(III)-Oxide

**DOI:** 10.3390/gels12050432

**Published:** 2026-05-15

**Authors:** Sanja Knežević, Marija Ivanović, Snežana Nenadović, Dorota Korte, Swapna Mohanachandran Nair Sindhu, Marijan Nečemer, Miloš Nenadović

**Affiliations:** 1 Department of Materials, Vinča Institute of Nuclear Sciences, National Institute of the Republic of Serbia, University of Belgrade, 11120 Belgrade, Serbia; 2 Institute of Nuclear Sciences “Vinča”, Mike Petrović Alasa12-14, Vinča, 11351 Belgrade, Serbia; 3 Laboratory for Environmental and Life Sciences, University of Nova Gorica, Vipavska 13, 5000 Nova Gorica, Slovenia; 4 Department of Optoelectronics, University of Kerala, Trivandrum 695581, India; 5 Department of Low and Medium Energy Physics, J. Stefan Institute, Jamova 39, 1000 Ljubljana, Slovenia; 6 Department of Atomics Physics, Vinča Institute of Nuclear Sciences, National Institute of the Republic of Serbia, University of Belgrade, 11120 Belgrade, Serbia

**Keywords:** aluminosilicate gels, samarium(III)-oxide, EDXRF, DRIFT, SEM, PBD

## Abstract

Aluminosilicate materials, known for their high strength, corrosion resistance, and thermal stability, are synthesized through the alkali activation of metakaolin, incorporating Sm_2_O_3_ to investigate the impact on their physicochemical properties. This study takes a look at the synthesis and physicochemical characterization of aluminosilicate gels doped with samarium(III)-oxide (Sm_2_O_3_), focusing on their potential as thermal insulators due to their enhanced thermal conductivity and absorption properties. Two samples with 1% and 5% Sm_2_O_3_ by weight were investigated, referred to as S_1_ and S_2_, respectively. Characterization techniques such as energy-dispersive X-ray fluorescence spectrometry (EDXRF), diffuse reflectance infrared Fourier transform spectroscopy (DRIFT), scanning electron microscopy (SEM), and photothermal beam deflection (PBD) were employed for the physicochemical characterization of aluminosilicate materials. The structural analysis shows an integration of Sm_2_O_3_, which did not significantly affect the gel’s density or porosity but enhanced its thermal conductivity. This study shows the potential of Sm_2_O_3_-doped aluminosilicates in applications requiring improved thermal management and stability, positioning them as potentially suitable materials for insulation.

## 1. Introduction

In the current era of extensive materials exploration, aluminosilicate materials have emerged as a category of inorganic polymer, garnering interest across materials science, engineering, and applied chemistry [1,2,3]. Aluminosilicate gels serve as a foundation for innovation in many industries, including construction, chemical, geotechnology, and environmental remediation. Their ability to offer exceptional properties, such as high strength, corrosion resistance, and thermal stability, makes them attractive for applications in various sectors, including construction, the chemical industry, geotechnology, and renewable energy sources. Research on aluminosilicate materials is continuously advancing by improving synthesis methods, understanding their microscopic structure, and expanding their applications to new fields like insulation, sustainable infrastructure, and energy storage [4]. These studies are essential for uncovering the potential of aluminosilicate gels as environmentally friendly options with enhanced performance. The key features of aluminosilicate gels stem from their chemical structure, which enables the formation of solid, high-density networks. During alkali activation, a cage-like structure forms within the aluminosilicate gels [5], allowing metal ions to be trapped [6] or participate in the activation process [7,8,9]. For example, incorporating samarium (Sm^3+^) ions into the aluminosilicate network significantly enhances their functional properties [7,10], including an increase in the specific surface area. This allows more interaction with the environment, improving the material’s efficiency as a photocatalyst because it absorbs UV radiation more effectively, thus boosting its ability to degrade organic pollutants or support cleaning processes [10,11,12]. Aluminosilicate materials also exhibit pozzolanic activity, and adding dopants can improve their durability and strength [13]. Moreover, aluminosilicate gels contain a high level of silicone combined with sodium-based activators and offer excellent thermal insulation, with the lowest thermal conductivity values achieved at specific porosity levels [14]. Aluminosilicate-based binders outperform traditional Portland cement binders due to their superior resistance to fire-induced cracking. Maintaining resistance to spalling is crucial for the structural integrity of buildings during and after fires, as it prevents explosive spalling under high heat [15]. Additionally, aluminosilicate materials help reduce the greenhouse effect by lowering CO_2_ emissions during alkali activation [16]. Research by Knežević et al. demonstrated that doping aluminosilicate gels with rare-earth elements alters their chemical and physical properties [17]. Saif et al. found that incorporating mixed powders into ultra-high-strength, fiber-reinforced, self-compacting concrete significantly improved its performance at elevated temperatures, highlighting its potential for sustainable use [18]. Similarly, our study showed that increasing Sm_2_O_3_ content resulted in notable changes in thermal conductivity, positioning these materials as promising candidates for energy-efficient structures and insulation applications.

During aluminosilicate gel synthesis, heavy metals can be incorporated, and stable phases can be formed with their incorporation into the aluminosilicate material [19]. Aluminosilicate materials exhibit pozzolanic activity that can be further increased during thermal treatment [20].

To improve the thermal conductivity of the base fluid, several researchers have used rare-earth oxide nanoparticles. Zirconium oxide, as a rare-earth oxide, is widely used in the ceramic industry, with a thermal conductivity of about 2 W/mK. Yttrium oxide has a thermal conductivity of 27 W/mK and is used in many applications due to its higher heat transport properties [21]. Selvan Pugalenthi et al. investigated the improvement in the thermal conductivity and stability of rare-earth metal oxide nanofluids using the stabilizing action of nano-CaCO_3_ in comparison with the stabilizing action of sodium dodecyl sulfate [22]. Also, by adding nanoparticles, researchers attempted to identify the mechanism underlying the increase in thermal conductivity. The mechanism behind the improvement of thermal conductivity with the addition of graphene nanoparticles to the base fluid was discussed by Jia-nan Huang et al. [23]. Artificial intelligence (AI)-based tools with different volume percentages (0.5 to 4.0%) and nanoparticle shapes in a nanofluid were used by Wei Cui et al. [24]. Qian Xu et al. analyzed the thermal performance of nanofluids containing TiO_2_ and Al_2_O_3_ nanoparticles in spherical and rod shapes [25]. In this way, they demonstrated an increase in thermal efficiency and heat transfer coefficients [25]. Suspended Al_2_O_3_ nanofluids in a shell-and-tube heat exchanger were used by Mehmet Senan Yılmaz et al. to improve the convective heat transfer coefficient [26]. While previous studies, such as Ivanovic et al. [27], investigated the structure and properties of metakaolin-based aluminosilicates, our research focused on the incorporation of different Sm_2_O_3_ concentrations (1% and 5%) and their effects on the physicochemical and thermal properties of the material. In contrast to previous studies [27,28], this study investigates how Sm_2_O_3_ doping affects thermal conductivity. Our findings show a direct correlation between Sm_2_O_3_ content and changes in thermal conductivity values, opening new possibilities for their application in thermal insulation. In addition, this study highlights the environmental benefits of using Sm_2_O_3_ as a chemical waste imitation in aluminosilicate production, which is in line with sustainability goals.

The existing literature primarily focuses on the structural and mechanical effects of rare-earth additions to aluminosilicates, such as their influence on strength, porosity, and chemical stability [6,7,17]. However, their impact on thermal conductivity has not yet been thoroughly investigated. Through an extensive characterization approach, this study provides new insights into the integration of Sm_2_O_3_ into the aluminosilicate network and its direct correlation with changes in thermal conductivity values. These results indicate that doping aluminosilicates with Sm_2_O_3_ may affect their thermal properties. Our research shows that these materials can be a good choice for thermal insulation and energy-efficient system applications, thereby contributing to the development of sustainable solutions and filling a gap in existing research. This research supports and advances the UN’s sustainable development goals, particularly SDG 9 (industry, innovation, and infrastructure), SDG 11 (sustainable cities and communities) by synthesizing new aluminosilicate materials for possible construction use and SDG 12 (responsible consumption and production) and SDG 13 (climate change) by using imitations of chemical waste (Sm_2_O_3_) and natural clay to produce aluminosilicate material, which reduces the greenhouse effect [29]. In accordance with the goals of increasing energy efficiency and reducing emissions in the construction sector, where thermal insulation plays a key role [30], developed materials doped with Sm_2_O_3_ show potential for application in modern insulation systems. While previous studies have broadly investigated the synthesis and characterization of aluminosilicate materials [27,31], limited research has focused on the influence of rare-earth doping [17], especially with Sm_2_O_3_, and on their thermal and structural properties. This study provides a novel approach to modifying aluminosilicate gels for enhanced performance. The novelty of this study is that it deals with the analysis of the role of Sm_2_O_3_ incorporation at different concentrations (1% and 5%) in metakaolin-based aluminosilicate gels. The significance of this research lies in its potential industrial applications, especially in the fields of thermal insulation, construction, and high-temperature-resistant materials. The addition of Sm_2_O_3_ changes the thermal conductivity but does not disrupt the structure of the material, making it potentially suitable for use in energy-efficient building materials and thermal management systems. From a sustainability perspective, this study contributes to the advancement of environmentally friendly materials.

## 2. Results and Discussion

### 2.1. Energy-Dispersive X-Ray Fluorescence Spectrometry (EDXRF)

Quantitative EDXRF chemical analysis determined the percentage composition of oxides in samples S_1_ and S_2_. The results show high levels of sodium oxide (Na_2_O), silicon(IV)-oxide (SiO_2_), aluminum(III)-oxide (Al_2_O_3_), and iron(III)-oxide (Fe_2_O_3_). Samples S_1_ and S_2_ exhibit good pozzolanic activity because the sum of silicon, aluminum, and iron oxides (SiO_2_ + Al_2_O_3_ + Fe_2_O_3_) exceeds 70%. For sample S_1_, it is 78.4%, while for sample S_2_, it is 77.49%. The results of the chemical analysis for both samples S_1_ and S_2_ are presented in Table 1.

The high sodium-oxide (Na_2_O) level occurs because of the activation solution used to initiate the alkali-activated reaction. The incorporation of samarium (III)-oxide (Sm_2_O_3_) was also confirmed, and sample S_2_ contains a higher content of samarium (III)-oxide, which was expected.

### 2.2. Density

The Archimedes method was used in this study to calculate the density of synthesized aluminosilicate gels containing 1% and 5% Sm_2_O_3_. First, the dry specimen was weighed, and its mass was denoted as m_1_. The specimen was then immersed in distilled water and boiled for 4 h to saturate the accessible open pores. After saturation, the apparent mass of the specimen while immersed in water was measured and denoted as m_2_. Finally, the saturated mass of the specimen measured in air was recorded as m_3_.

The exterior volume of the specimen, V_ext_, was calculated according to Archimedes’ principle as

$$v_{ext}=\frac{m_{3}-m_{2}}{\rho_{w}}$$

where ρ_w_ is the density of water.

The bulk density, ρ_b_, was calculated as

$$\rho_{b}=\frac{m}{v_{e\times t}}=\frac{m_{1}}{m_{3}-m_{2}}\rho_{w}$$

The apparent porosity, P_a_, was calculated using

$$P_{a}(\%)=\frac{m_{3}-m_{1}}{m_{3}-m_{2}}\times 100$$

The results are shown in Table 2.

The density determination results indicate a relatively high density, suggesting that the material is compact yet has a significant open porosity of 29.04%. Open porosity refers to interconnected and open pores to the outside, allowing liquids and gases to pass through the material. A high open porosity value can be helpful in applications where the material’s permeability is required, such as filtration systems or insulation materials.

The density of the S_2_ sample, 1.4696 g/cm^3^, is similar to that of sample S_1_, indicating that the addition of 5% Sm_2_O_3_ did not significantly change the material’s density. This suggests that Sm_2_O_3_ is well integrated into the material matrix without significantly increasing the density.

The 29.12% porosity is very close to the sample containing 1% Sm_2_O_3_, indicating that the addition had no significant effect on the material’s pore structure.

Adding 5% samarium(III) oxide (sample S_2_) to the gel caused only minor changes in density and porosity, indicating that Sm_2_O_3_ was successfully incorporated into the gel’s structure. Interestingly, this integration could still boost certain properties of the material. Depending on how samarium(III) oxide is distributed within the matrix, it might enhance features like high-temperature resistance, electrical conductivity, and magnetic properties.

### 2.3. Diffuse Reflectance Infrared Fourier Transform Spectroscopy Analysis (DRIFT)

**Figure 1:**
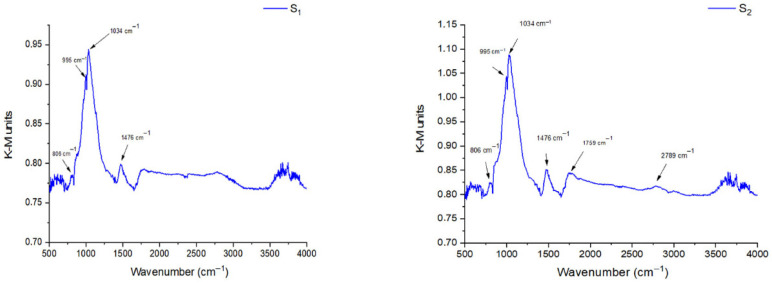
DRIFT spectra of samples S_1_ and S_2_.

The Kubelka–Munk function was used to analyze DRIFT spectra and identify the presence of various chemical bonds and groups in the material. According to the bands identified in the spectra shown in Figure 1 the band around 1034 cm^−1^ is associated with phonon scatterings of Si-O-Si (siloxane) or Si-O-Al bonds within the aluminosilicate material [17]. However, the Sm^3+^ ion can also be present during phonon scattering, leading to overlapping absorption bands [32].

The bands at 995 cm^−1^ correspond to the asymmetric stretching of Si-O bonds in the aluminosilicate matrix [33]. The shift in the position of these bands between the S_1_ and S_2_ spectra may suggest changes in the local environment of silicon centers caused by varying amounts of samarium oxide. The band at 806 cm^−1^ may be associated with phonon scatterings of Al-O-Si bonds, where aluminum is arranged in tetrahedral orientations [6].

The presence of carbonate groups is possible, and characteristic phonon scattering occurs at 1476 cm^−1^. The material is likely carbonized due to exposure to air [17]. The band at 2789 cm^−1^ is uncommon for inorganic compounds, suggesting that there may be some form of organic contamination in the material [34].

### 2.4. SEM/EDS Analysis

**Figure 2:**
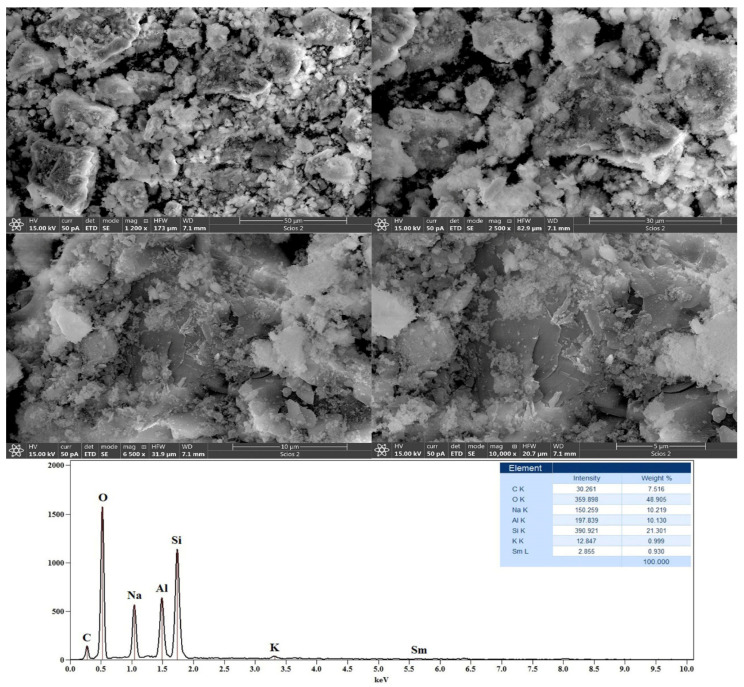
SEM micrographs and EDS analysis of sample S_1_.

**Figure 3:**
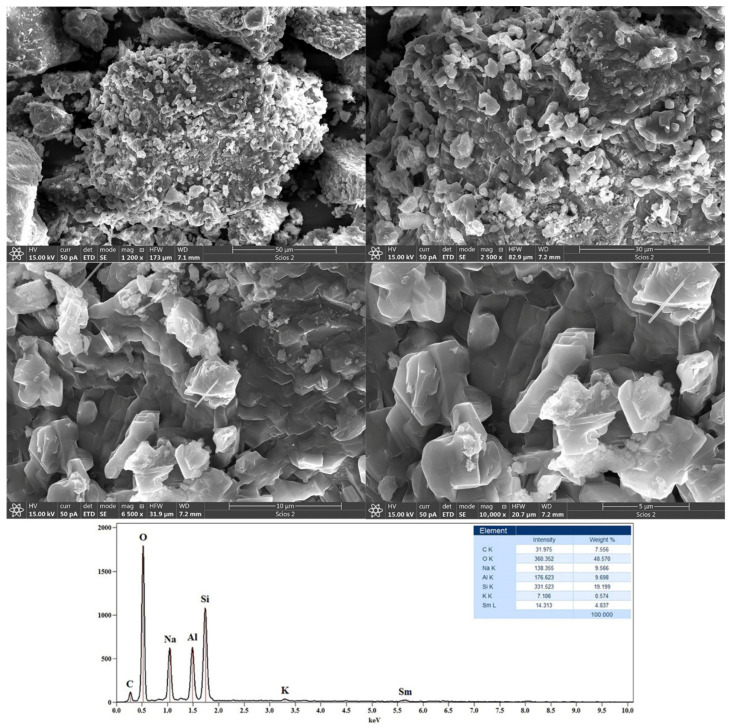
SEM micrographs and EDS analysis of sample S_2_.

SEM and EDS analyses of the synthesized samples are shown in Figure 2 and Figure 3. Scanning electron microscopy produces micrographs that help examine the morphology of the matrix itself. The material’s texture is heterogeneous, featuring dense and inter-particle voids, but in some parts of the sample, there is visible inhomogeneity in the distribution. This may be due to factors such as the initial mixing process, the reactivity of the metakaolin, or the curing conditions. The used magnifications were 1200×, 2500×, 6500×, and 10,000×.

For the sample S_1_ in Figure 2, the SEM micrographs reveal different particle sizes, with some larger particles possibly being partially reacted metakaolin, while the smoother areas could represent the aluminosilicate gel that acts as a binder. Depending on its reactivity, samarium(III)-oxide may be dispersed throughout the matrix or concentrated in specific regions, influencing the local microstructure. The presence of finer particles suggests a more complete alkali activation of the material. The spaces observed between material clusters are characteristic of the inter-particle arrangement in such matrices. Overall, the SEM analysis at different magnifications shows that adding 1% Sm_2_O_3_ affects the microstructure of the aluminosilicate material, potentially impacting its physical properties, such as mechanical strength and chemical resistance. EDS analyses confirmed the incorporation of 1% Sm_2_O_3_, with a higher presence of Al, Si, and Na compared to other elements, as further verified by EDXRF analysis.

EDS and EDXRF analyses also match for sample S_2_, and the results are shown in Figure 3. The first image provides a broad view of the aluminosilicate surface. The heterogeneous nature is clear from the rough textures and varying particle sizes. The large agglomerates exhibit higher brightness, which could be attributed to the higher atomic number of Sm^3+^, resulting in stronger backscatter electron contrast and suggesting localized Sm-rich regions. The finer particles likely represent the primary aluminosilicate matrix.

At 2500× magnification, the distinction between individual particles becomes more apparent. A variety of shapes and sizes can be observed, indicating a non-uniform reaction. The rough surfaces of the aggregates are typical of aluminosilicate processes in which the alkaline activator dissolves the aluminosilicate precursor particles, forming a gel that later solidifies. At 6500× magnification, the increased detail provides insights into particle bonding and may highlight areas where alkali activation is more or less complete. At the highest magnification, the focus shifts to individual particle interactions and the finer texture of the material. The particles display sharp edges, typical of crystalline phases. The presence of samarium(III) oxide is assumed based on the brighter particles, which are consistent with the electron backscatter contrast, where heavier elements appear brighter. EDS analysis confirmed the incorporation of 5% Sm_2_O_3_.

From Figure 2 and Figure 3, we can infer that the addition of Sm_2_O_3_ influences the alkali activation process, likely affecting the microstructure and potentially the mechanical properties of the material. The images demonstrate a complex interaction between the metakaolin and Sm_2_O_3_, resulting in a diverse microstructure with regions of different textures and particle integration. The particle morphology and the arrangement of voids observed across the magnifications play a role in defining the mechanical strength, durability, and chemical resistance of the aluminosilicate material.

### 2.5. The Home-Built Photothermal Beam Deflection (PBD)

By analyzing thermal conductivity, it was found that sample S_1_ has a value of 0.41 W/mK, while sample S_2_ has a higher value of 0.69 W/mK. These values are shown in Table 3. To contextualize the thermal conductivity of samples S_1_ and S_2_, the results were compared with data from the literature.

In a study by Waleed et al., the thermal conductivity range for ordinary cement and lightweight concrete is between 0.4 and 2.5 W/mK [35]. The values of our material fall within this range. The specific values indicate that the aluminosilicate matrix is likely quite porous, resulting in lower thermal conductivity.

A higher concentration of Sm_2_O_3_ in sample S_2_ can lead to increased thermal conductivity. The significant difference in thermal conductivity between the two samples shows that adding Sm_2_O_3_ has an effect on the material’s heat conduction capabilities. Since S_2_’s thermal conductivity is higher, this suggests that Sm_2_O_3_ influences the structure and may also interact with the aluminosilicate matrix.

Thermal conductivity is an important property for materials that might be used in thermal insulation, electronic substrates, or any application where heat transfer is critical. The lower thermal conductivity of S_1_ suggests that it might be better suited for insulation purposes, while S_2_, with its higher conductivity, could find use in applications where some heat transfer is desirable. To better understand the performance of the developed materials, the thermal conductivity of sample S_2_ (0.69 W/mK) was compared with typical values for commercial materials. Although higher than values for insulating materials like rock wool (~0.035–0.045 W/mK) [36] and polyurethane foam (~0.020–0.030 W/mK) [37], it is significantly lower than the thermal conductivity of ordinary silicate concrete (~1.4–1.8 W/mK) [36]. Additionally, the value of sample S_2_ falls within the range of lightweight concrete (~0.3–0.6 W/mK) used in energy-efficient construction [35]. This suggests that Sm_2_O_3_-doped aluminosilicate gel could serve as a medium-conductivity thermal insulating material, suitable for applications requiring a balanced combination of mechanical strength and insulating properties.

## 3. Conclusions

This study examines the synthesis, physicochemical characterization, and assessment of thermal properties of alkaline-activated materials doped with samarium(III)-oxide (Sm_2_O_3_). By adding Sm_2_O_3_ into the aluminosilicate matrix at concentrations of 1% and 5% by weight, we sought to explore its effects on the material’s structure and function. Our research confirms the integration of Sm_2_O_3_ into the aluminosilicate matrix, as evidenced by energy-dispersive X-ray fluorescence spectrometry (EDXRF), which detected a significant amount of samarium in the samples. Structural analyses using diffuse reflectance infrared Fourier transform spectroscopy (DRIFT) and scanning electron microscopy (SEM) provided insights into the microstructural changes caused by Sm_2_O_3_ doping. Thermal conductivity measurements revealed a slight increase in conductivity with higher Sm_2_O_3_ concentrations. This suggests that the dopant integrates effectively into the matrix, potentially leading to a more continuous structural network or localized densification, without significantly compromising the material’s inherent low porosity. While thermal conductivity increased slightly, the values remain within the range suitable for specialized thermal management applications. Our research shows that Sm_2_O_3_-doped aluminosilicate gels preserve the benefits of aluminosilicates, such as their high strength and durability, while also exhibiting tailored thermal stability. These results highlight the potential of Sm-doped aluminosilicates as sustainable, multifunctional materials for industrial applications in extreme environments. Future studies will explore a wider range of Sm_2_O_3_ concentrations and assess the material’s long-term durability to better understand its thermal and structural stability.

## 4. Materials and Methods

Metakaolin, obtained by calcining kaolinite ore (Rudovci near Lazarevac, Republic of Serbia), was used as the starting material. Metakaolin reacts with an alkaline activator and forms an aluminosilicate material. The alkaline activator is a mixture of sodium hydroxide (pro analysi, 1 kg, NRK engineering (Tamil Nadu, India), CAS: 1310-73-2) and sodium silicate solutions (aqueous solution of Na_2_SiO_3_, resistant to 800 °C, Interhem Company (Białystok, Poland)). In this work, a 12 M sodium hydroxide solution was used, while the volumetric ratio of the solution was 1.5 (Na_2_SiO_3_/NaOH = 1.5). During the alkali activation process, commercially available Sm_2_O_3_ powder was mixed during the process of alkali activation in aluminosilicate gel. The aluminosilicate samples were prepared using two samarium (III)-oxide (>99, 10 g, Merck KgaA (Darmstadt, Germany), CAS: 12060-58-1) weight fractions (1% and 5%), and named S_1_ and S_2_. After the alkali activation process, samples were left at room temperature for a day. The mixture was kept in an oven at 60 °C for two days in covered molds, and then, it matured at room temperature under regulated conditions for 28 days.

### 4.1. Energy-Dispersive X-Ray Fluorescence Spectrometry (EDXRF)

EDXRF is a fast, simple, and cheap non-destructive multielement analytical technique. The pulverized sample was pressed into a pellet and then directly analyzed by an in house bulit EDXRF spectrometer using three monochromatic excitation sources of Fe-55, Cd-109, and Am-241. This excitation configuration enables optimal excitation from light elements (Na and K) to heavier elements (Nd and Sm). The emitted fluorescence radiation was measured using the EDXRF spectrometers with SDD or Si (Li) detectors. The details of the applied spectrometer configuration and quantitative analysis software are in references [38,39].

### 4.2. Diffuse Reflectance Infrared Fourier Transform Spectroscopy (DRIFT)

Diffuse reflectance infrared Fourier transform spectroscopy (DRIFT) was used for analyzing the aluminosilicate material. Drift spectra were collected by a Perkin-Elmer FTIR spectrometer (PerkinElmer, Shelton, CT, USA). About 5% of the samples were dispersed in oven-dried spectroscopic grade KBr with a refractive index of 1.559 and particle size of 5–20 µm. Background KBr spectra were acquired and ratioed to the background. The spectra were scanned at 4 cm^−1^ resolution and collected in the mid-IR range (4000–400 cm^−1^).

### 4.3. Scanning Electron Microscopy (SEM)

An emission scanning electron microscope (FESEM, FEI Scios 2, Dual Beam system, Thermo Fisher Scientific, Waltham, MA, USA) was used for morphological analysis. The samples were attached to the sample holder with double-sided copper tape. Micrographs were collected at an accelerating voltage of 15 kV and a chamber pressure of about 9 × 10^−5^ Pa. The magnification used in the work was 1200×, 2500×, 6500×, and 10,000×.

### 4.4. The Home-Built Photothermal Beam Deflection (PBD)

**Figure 4:**
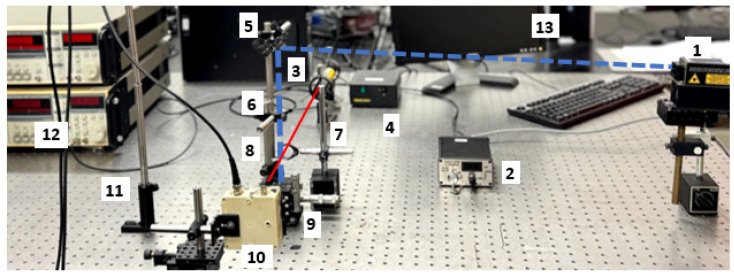
1. Modulated EB; 2. EB controller; 3. PB; 4. PB controller; 5. beam splitter; 6. EB forming lens; 7. PB forming lens; 8. sample holder; 9. 3D translation stage; 10. quadrant photodiode; 11. EB modulation; 12. lock-in amplifier; 13. PC.

**Scheme 1:**
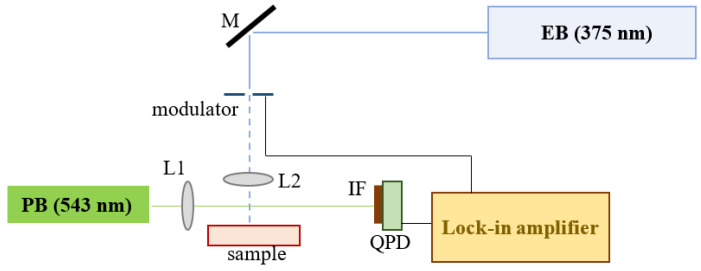
EB—excitation (pump) beam; PB—probe beam; L1; L2—lenses; M—mirror; IF—interference filter; QPD—quadrant photodiode (detector).

The home-built photothermal beam deflection (PBD) setup was used to evaluate the thermal conductivity of the samples, as shown in Figure 4. The excitation beam (EB) is generated by a solid-state laser (Oxxius S.A., LBX-445-500-CSB-PPA, Lannion, France), providing 80 mW of output power at 375 nm, while the probe beam (PB) source is a He-Ne laser (Melles Griot, 25-LGR-393-230, Carlsbad, CA, USA) (2 mW, 543.5 nm). The EB is modulated directly from the internal oscillator of an SR-830 lock-in amplifier (Stanford Research Systems, Inc., Sunnyvale, CA, USA) within a 5–220 Hz frequency range and shaped by a lens with 10 cm focal length (Bi-Convex, AR-Coated: 350–700 nm), as shown in Scheme 1. It is further directed perpendicularly onto the sample surface placed on an XYZ translator stage, which allows the experimental setup to be easily adjusted. As a result of the optical absorption of EB by the sample and conversion of optical energy into heat, temperature oscillations (TOs) are induced in the sample and its surroundings [40]. Another Bi-Convex lens (AR-Coated: 350–700 nm) of 5 cm focal length is used to focus PB over the sample and align it in such a way that it skims the sample’s surface, crossing EB perpendicularly to the direction of its propagation. A visible quadrant cell photoreceiver (Newport 0901 with New Focus photodetector head Model 2901, Newport Corporation, Irvine, CA, USA) coupled with a 532 nm interference filter (Edmund Optics, Barrington, NJ, USA) is used to detect and measure deflections caused by the interaction of PB with the photo-induced TOs.

The variations in the phase and amplitude are recorded as a function of modulation frequency. To extract the thermal properties of the analyzed sample from the experimental data, a previously developed multi-parameter fitting procedure was used [41,42]. The fitting procedure was performed according to the protocol described in the literature [43]. The experimental setup was optimized to ensure the maximum sensitivity of measurements, as described in [44]. The amplitude and phase of the photodeflection signal were measured as a function of the modulation frequency of the excitation radiation. The thermal properties of the examined samples were extracted from the experimental data (amplitude and phase of the BDS signal) by the use of a least-squares procedure. The fitted parameters were the radius of the probe beam, its waist position and height over the sample surface, and the thermal conductivity of the examined sample. The RSD (relative standard deviation) of the determined properties does not exceed 5%.

## Figures and Tables

**Table 1:** EDXRF major elements quantitative analysis of oxides of the investigated samples.

Oxide	Sample	
	S_1_ (%)	S_2_ (%)
Na_2_O	16.95	14.33
MgO	1.81	3.17
Al_2_O_3_	23.76	24.19
SiO_2_	53.11	51.89
K_2_O	1.55	1.53
CaO	0.29	0.29
TiO_2_	0.26	0.21
Fe_2_O_3_	1.53	1.41
Sm_2_O_3_	0.63	2.89

**Table 2:** Density of samples S_1_ and S_2_.

Sample	m_1_ (g)	m_2_ (g)	m_3_ (g)	Exterior Volume, V_ext_ (cm^3^)	Bulk Density, ρ_b_ (g/cm^3^)	Apparent (Open) Porosity P_a_ (vol %)
S_1_	1.1453	0.5885	1.3732	0.7847	1.4595	29.04%
S_2_	1.6797	0.8695	2.0125	1.1430	1.4696	29.12%

**Table 3:** Thermal conductivity of samples S_1_ and S_2_.

Sample	Thermal Conductivity (W/mK)
S_1_	0.41
S_2_	0.69

## Data Availability

Data are contained within the article.
